# Is There a Link between COVID-19 Infection, Periodontal Disease and Acute Myocardial Infarction?

**DOI:** 10.3390/life11101050

**Published:** 2021-10-07

**Authors:** Ioana-Patricia Rodean, Carmen-Ioana Biriș, Vasile-Bogdan Halațiu, Andrei Modiga, Luminița Lazăr, Imre Benedek, Theodora Benedek

**Affiliations:** 1 Clinic of Cardiology, Emergency Clinical County Hospital, 540136 Târgu Mureș, Romania; ioana.rodean@umfst.ro (I.-P.R.); imrebenedek@yahoo.com (I.B.); theodora.benedek@gmail.com (T.B.); 2 Center of Advanced Research in Multimodality Cardiac Imaging, CardioMed Medical Center, 540124 Târgu Mureș, Romania; 3 “George Emil Palade” University of Medicine, Pharmacy, Science and Technology, 540139 Târgu Mureș, Romania; biriscarmen74@yahoo.com (C.-I.B.); modiga.andrei94@gmail.com (A.M.); luminita.lazar@umfst.ro (L.L.); 4 UPU-SMURD, Emergency Clinical County Hospital, 540136 Târgu Mureș, Romania

**Keywords:** acute coronary syndrome, COVID-19 infection, cytokine storm, inflammation, miRNA, periodontal disease

## Abstract

Both periodontal disease and atherosclerosis are chronic disorders with an inflammatory substrate that leads to alteration of the host’s immune response. In PD, inflammation is responsible for bone tissue destruction, while in atherosclerosis, it leads to atheromatous plaque formation. These modifications result from the action of pro-inflammatory cytokines that are secreted both locally at gingival or coronary sites, and systemically. Recently, it was observed that in patients with PD or with cardiovascular disease, COVID-19 infection is prone to be more severe. While the association between PD, inflammation and cardiovascular disease is well-known, the impact of COVID-19-related inflammation on the systemic complications of these conditions has not been established yet. The purpose of this review is to bring light upon the latest advances in understanding the link between periodontal–cardiovascular diseases and COVID-19 infection.

## 1. Inflammation, Atherosclerosis and Acute Coronary Syndromes

Acute coronary syndromes (ACS) are defined as major cardiovascular emergencies usually triggered by a sudden decrease in the blood flow due to an acute occlusion of the coronary arteries, which may occur from plaque disruption and/or thrombotic occlusion [1,2,3]. ACS remains a major cause of death in developing countries, accounting for almost 1.8 million deaths annually (totaling 31% of all deaths worldwide, respectively 20% of those registered in Europe). Among the manifestations of ACS, myocardial infarction (MI) is still the leading cause of morbidity and mortality worldwide. Furthermore, 85% of deaths caused by cardiovascular disease (CVD) are due to MI, accounting for about one-third of deaths in patients over 35 years of age [2,3,4,5,6]. It is also well known that various medical conditions, such as respiratory failure or infectious diseases can lead to an imbalance between oxygen demand and supply, thus favoring the occurrence of myocardial ischemia (type 2 MI) [3].

The aim of this article is to provide a better understanding of the inflammatory triangle that underlies acute myocardial infarction (MI), periodontal disease (PD) and COVID-19 infection. We searched PubMed, Google Scholar, Web of Science and EBSCOhost, four frequently used databases by researchers. We started the literature search by using the keywords “acute coronary syndrome”, “COVID-19 infection”, “cytokine storm”, “inflammation” and “periodontal disease”. Papers published in the last 15 years were included in the present article if the content seemed to discuss the methodology of the literature review process.

Inflammation represents the key mechanism of atherosclerosis, and several clinical trials tried to demonstrate the role of inflammation in the atherosclerotic process. The CANTOS trial, including 10.061 patients who have previously suffered an MI, reported a significant reduction in serum levels of the high-sensitivity C-reactive protein (hsCRP) after administration of the anti-inflammatory canakinumab, with 26% for the group with 50 mg canakinumab, 37% for those with 150 mg and 41% in the 300 mg group. Moreover, inhibition of interleukin (IL) 1B significantly reduced the risk of major cardiovascular events (MACE) [7].

At the same time, plaque progression and instability are sustained and accelerated by different degrees of inflammation [4,7,8,9,10,11]. Various studies have shown that atherosclerotic lesions contain a series of pro-inflammatory cytokines (IL 1, 6, 8, tumor necrosis factor), cells that favor the formation of the fibrous cap [12,13]. A vulnerable plaque (VP) is defined as an atheromatous lesion that suffered phenotype changes that makes the plaque more prone to rupture. The typical VP contains a thin fibrous cap and a large lipid core, and has distinct features at CT examination, such as the “napkin ring” sign, spotty calcification, positive remodeling or low attenuation grade. The presence of vulnerability features associated with a pro-inflammatory status, caused by the secretion of pro-thrombogenic molecules, collagen, metalloproteinase, tissue factor, vascular and molecular adhesion molecules, expressed on the plaque surface, lead to a predisposition of the patient to develop an ACS through complete obstruction of the vascular lumen by a thrombus. In more than 75% of cases, it was proved that the MI appears due to a coronary plaque rupture [14,15].

The pathogenesis of atherosclerosis is a very complex mechanism, involving several risk factors, most of them common with other diseases, especially with the inflammatory ones. Periodontal disease (PD) is one of the most frequent inflammatory condition associated with CVD, with a possible direct implication in the plaque instability process, due to the oral pathogens and through the cascade of inflammation that it activates.

## 2. Periodontal Disease—The Local Inflammatory Disease Increasing Cardiovascular Risk

In the past 5 years, an increased incidence of the PD has been observed within the young population, currently being in the top ten most prevalent diseases among youngsters in the world [2,4]. In the USA, almost 50% of the population over the age of 30 have PD and this percentage rises to almost 60% in people under 65 years old, about 9% of them having a severe form of PD [2,5,16]. PD is characterized by a chronic inflammatory condition produced by the alteration of the normal oral microbiota and is depicted as the destruction of the soft and hard structures supporting the teeth, due to repeated bacterial infections [2,8]. According to the severity of the PD, this condition can be classified in two major stages of evolution. The first one is gingivitis, which occurs in the early stages of PD, being characterized by oedema and redness of the gum. The second one is periodontitis, which occurs in the advanced stages of PD and is defined by the destruction of the supporting structurers of the teeth, leading to their loss [2].

Associated comorbidities or cardiovascular risk factors, such as diabetes, hypercholesterolemia, smoking habits, obesity or stress have been described in patients with PD [2,17]. Moreover, it was observed that these risk factors are more frequent in patients with severe forms of COVID-19 infection, a viral infection with a strong inflammatory substrate [18]. It was also demonstrated that several oral pathogens are directly involved in the onset of atherosclerotic process, and recent studies have demonstrated a strong link between PD and ACS. [5,17]. It seems that the more advanced the PD, the greater the risk of developing an ACS [2,17]. The pathophysiological substrate of this link is represented by the initiation and maintenance of the inflammatory cascade, both locally (at gingiva site) and systemically, triggered by local inflammation at the level of periodontal plaque.

Considering that not all the patients with PD develop severe forms of CVD, the hypothesis of genetic susceptibility to a common pathophysiological link between PD and CVD has been raised. Both PD and CVD trigger the inflammatory cascade, based on the interaction between pro-inflammatory and pro-thrombotic cytokines, favoring progression of the inflammatory syndrome that started from a gingival infection.

A study published by Wojtkowska et al. (2020) conducted on 111 patients showed a significant correlation between advanced PD and ACS (*p* < 0.05). Moreover, from all periodontal indices, plaque accumulation and gingival bleeding are the ones more highly corelated with ACS (*p* < 0.001 and *p* = 0.001) [19]. Similar results were obtained by Gomes-Filho et al. (2020), who proved that the PD severity is strongly associated with the risk of developing an ACS (two to four times higher risk) [20]. Similar results were obtained in the ATHERODENT trial, an Angio-CT based study that investigated the correlation between PD and CT features of vulnerable atheromatous plaques, and revealed a direct link between PD severity, atherosclerosis and coronary plaque vulnerability in patients with unstable angina (UA) [(21]. Thus, it was observed that in patients with high-risk plaques (that contain at least two of the following vulnerability features: napkin ring, spotty calcification, low attenuation atheroma and positive remodeling), the PD was more severe compared with those without VP (28.20 ± 13.34 vs. 18.71 ± 11.31, *p* = 0.001). Moreover, a higher calcium score was identified in patients with PD, especially in those with severe forms (*p* = 0.0001). The gingival indices most frequently associated with ACS in the ATHERODENT trial were papillary bleeding index (4.5 ± 3.06 vs. 2.04 ± 1.96, *p* = 0.002) and clinical attachment loss, an irreversible sign of PD (3.6 ± 2.91 vs. 1.66 ± 1.8, *p* = 0.009) [21].

Several studies investigated the role of different oral microorganisms involved in PD. Daily dental activities, as well as repeated bacteremia, favor the invasion of dental microorganisms into the systemic circulation and can cause local inflammatory reactions, such as those located at the level of coronary atheroma [22].

Gram-negative bacteria are the microorganisms most frequently involved in PD pathogenesis [17]. From all these bacteria, *P. gingivalis* is the most commonly found in patients with PD (67%) and is responsible for the secretion of cytokines and cell adhesion molecules, including intercellular and vascular cell adhesion molecules (ICAM, VCAM) [17,22,23]. Additionally, a study published in 2019 showed that *P. gingivalis* stimulates the cell proliferation and produces endothelial dysfunction [23].

At the level of the atheromatous plaques, DNA fragments from *Chlamydia pneumoniae*, *Enterococus fecalis*, *Prophromonas endotelialis* and *Streptococcus* species have been identified [22]. A study conducted by Joshi et al. (2020) proved that *P. gingivalis* was the bacteria most frequently found in atheromatous plaques (*p* = 0.00003) [22]. Moreover, the presence of this oral pathogen was associated with development of ACS (*p* = 0.096) [24]. Similar results were obtained by Xie et colab. (2020), who demonstrated that *P. gingivalis* promotes and accelerates the atherosclerotic process. The mechanism involved in this process seems to be represented by an NF-kB-BMAK1-NF-kB signaling loop [25]. However, a study conducted by Schlutz et al. (2020) proved no correlation between various dental pathogens (including *P. gingivalis*) and CVD. Still, *E. corrodens* was found to be associated with a lower risk of developing recurrent MACE (*p* = 0.001) [26].

The literature data showed that the use of nucleic acid sequencing technologies (NASt) provides superior results in the examination of normal and pathogenic oral microbial flora [27]. Studies using the NASt revealed that *P. gingivalis*, *T. denticola* and *T. forsythia* are the most frequent pathogens (red/orange complex species) associated with severe forms of PD [28]. Future studies aim to predict the occurrence of CVD related to the oral microbiome using artificial intelligence, in the hope that the link between the microbiome and CVD can be explored using AI [29].

## 3. Inflammatory Biomarkers in Cardiovascular Disease and Periodontal Disease

The host cell’s response against bacterial pathogens leads to an excessive and continuous production of inflammatory mediators with an important role in tissue destruction [4].

The most widely used biomarker of systemic inflammation is CRP. Its serum levels begin to increase from the earliest stages of PD, and it has the role of capturing the IL 1 to IL 6 inflammatory axis up-stream [30]. The CRP role in atherogenesis is well known, namely its ability to bind to low-density lipoproteins promoting endothelial disfunction and plaque instability [31]. Research has shown that in patients with associated PD and CVD, the CRP level is significantly higher compared to those with PD without CVD; therefore, it can be considered that the CRP is a cardiovascular risk factor [31,32]. Moreover, a study conducted by Wojtkowska et al. (2020) revealed that hsCRP is positively correlated with periodontal markers; therefore, with severe forms of PD (*p* < 0.05) and with ACS [19]. A study performed by Torrungruang et al. (2018) on 799 patients, revealed that the severity of the PD is directly correlated with the seric level of sST2 and hsCRP (*p* < 0.05) [33]. Similar results were obtained in a randomized clinical trial conducted by Montenegro et al. (2018). Therefore, it was shown that in patients with seric CRP level > 3 mg/L, a significant reduction was obtained after 3 months of PD treatment in the tested group compared to the control one (*p* = 0.04). Moreover, in the same trial, it was observed that the IL 6 and IL 8 levels after 3 months were lower in patients with PD treatment compared to the control group [34].

Another relevant pro-inflammatory cytokine involved in the link between PD and CVD, is represented by IL 1B [4]. A study conducted by Diaz et al. (2020), performed on 260 subjects, showed that a higher percentage of subjects present both conditions (ACS and PD), but without a significant correlation regarding the IL 1B serum level in patients with ACS with or without PD. However, in all groups of subjects the serum level of IL 1B was higher compared to the healthy ones. It has also been shown that in patients with ACS, the CRP, serum levels of IL-6 are rising directly proportional with the PD severity and are more expressed in patients with ACS and PD [4,32]. Additionally, it was observed that IL 6 is an independent risk factor for cardiovascular events (OR, 1.6; 95% CI, 1.4–1.8) [35].

Another important cytokine involved in the pathogenesis of both diseases is matrix metalloproteinase (MMP). In PD, MMP is related with connective tissue destruction, while in CVD it accelerates the atherosclerotic process and leads to plaque instability [31,32]. Recent findings proved that MMP 8 is more expressed in patients with chronic PD compared to healthy ones [32]. In patients with both ACS and PD, it was pointed out that the serum levels of MMP8 and MMP9 were higher compared to the control group, but nevertheless, after PD adjustment, the levels of these cytokines failed to demonstrate an association with ACS [31].

It was assumed that the link between PD and CVD is best expressed by periostin (Pn), a novel inflammatory biomarker released from the level of dental structures. Recent studies have shown that Pn is involved in a reduction in infarction size, decrease in fibrosis, improving ventricular remodeling [36]. A study performed by Oka et al. (2007), on mice, revealed that Pn is very important in the healing process, after an ACS. Their results demonstrated that mice without the Pn gene coding were more susceptible to cardiac rupture after 2 weeks from the acute event (*p* < 0.05), compared to Pn mice [37]. Similar results were found in a study conducted in Japan on rats who received post-MI neutralizing Pn antibodies. It was observed that not only the infarcted area was reduced (*p* < 0.05), but also the left ventricular function was improved after 8 weeks from the acute event (*p* < 0.01) [38]. Furthermore, in a study conducted by Chen et al. (2017), it was shown that in neonatal mice, the Pn level was significantly increased at 1, 3 and 7 days after an ACS (*p* < 0.01) [39]. Recent data also proved that Pn is involved in the atherosclerosis process and its serum level is increased in advanced forms of heart failure or ACS [40].

In a large case control study performed on 990 patients, it was described that in the group with CVD, the Pn level was increased compared to the control group (*p* < 0.005) [41]. Nevertheless, in a study conducted by Cheng et al. (2012), it was concluded that the Pn level was significantly lower in patients with ACS (*p* < 0.001) [42]. The results from another study conducted in China by Ling (2014) showed that the Pn level is positively associated with the Killip class in patients with ST-elevated MI (*p* < 0.01). Additionally, in patients with ST-elevation MI, the Pn level was negatively correlated with the left ventricular function and more frequently associated with the left anterior descending artery restenosis [43]. Furthermore, recent data concluded that Pn is significantly correlated not only with N-terminal b-type natriuretic peptide but also with the sensitive troponin and ST2 [44].

As described previously, Pn molecules are believed to be a future therapy and a novel biomarker for patients with MI, due to its important role in the regeneration and healing of the myocardial muscle [36,45].

## 4. COVID-19—The New Trigger of Systemic Inflammation

COVID-19 infection was defined as a challenging inflammatory disease produced by a coronavirus, being characterized by a clinical polymorphism, which can lead to a severe acute respiratory syndrome (SARS) [46]. The rapid spread of this disease has led to a global pandemic and has raised the urgent need of developing a risk scale for patients infected with SARS-CoV-2 [47].

At the same time, various inflammatory parameters proved to be directly associated with the severity of COVID-19 disease. Serum levels of CRP, a marker of systemic inflammation, increase from the early stages of infection, and recent data proved that CRP level is positively associated with CT severity of COVID-19 infection (*p* < 0.01) [47]. Additionally, it was seen that in patients with COVID-19 infection, the level of Matrix metalloproteases-9 is higher, being significantly associated with neutrophil count (*p* < 0.001) [48]. In a study conducted by Jorgensen et al. (2020), it was demonstrated that the level of pro-inflammatory cytokines is increased in patients with COVID-19 infection, and their serum level is directly correlated with the severity of infection. In all 34 patients enrolled in this study, IL6 was higher and was associated with severe forms of respiratory failure (*p* < 0.01) [49].

Starting from the premise that in COVID-19 infections, the mechanism of action is represented by the ability of the virus to bind to angiotensin-converting enzyme 2 (ACE2) receptors, it was assumed that the occurrence of multiple organ dysfunction is strongly associated with COVID-19 due to the wide distribution of ACE2, in several organs—lungs, kidney, esophagus, vessels [47]. Moreover, another possible mechanism of action described was vasculitis and endothelial damage. In both situations, the inflammatory cascade and the cytokine storm are activated, leading to a systemic inflammatory state. All these changes play an important role in CVD pathophysiology and may explain the link between CVD and COVID-19 infection [47,50].

The results of the most recent studies about the role of inflammatory biomarkers in COVID-19 disease are presented in Table 1.

Several studies have proven a direct link between the severity of the SARS-COV2 infection and the occurrence of MI. Thus, Huang et al. showed that from 41 patients admitted in hospital with pneumonia and abnormal CT pulmonary findings, 12% have developed myocardial injury and one-third of them needed admission in the intensive care unit (ICU). Moreover, ICU patients presented higher levels of pro-inflammatory and pro-thrombotic molecules, such as IL, TNF, etc., in their plasma. [64]. In a larger study, conducted by Wang et al., from 138 patients hospitalized for COVID-19 infection, 26% were treated in the ICU due to severe complications, including MI [65]. Interesting results were found by Zhou et al., in a study in which from almost 200 patients with COVID-19 infection, 17% developed an AMI, and non-survivors were more prone to develop MI compared to survivors (32.5% vs. 1%) [66]. The main results of the major studies regarding mortality in COVID-19 patients with and without myocardial injury are presented in Table 2.

## 5. The Inflammatory Link between Periodontal Disease, Acute Coronary Syndrome and COVID-19 Infection

**Figure 1:**
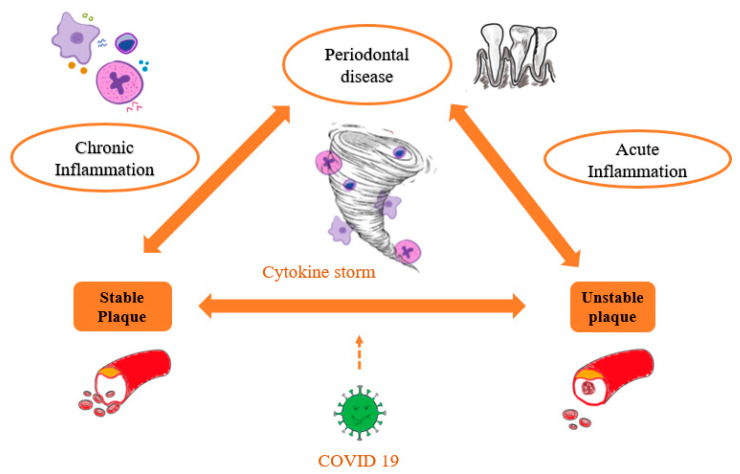
The link between periodontal disease, cardiovascular disease and COVID-19.

Starting from the premise that in PD, CVD and in the COVID-19 infection, a series of pro-inflammatory cytokines are released into the bloodstream, the possibility of a link between severe forms of COVID-19, PD and CVD has risen (Figure 1). Several risk factors are shared by these three diseases, and it was proven that patients with comorbidities develop more severe forms of COVID-19 [18]. The action of different bacterial species expressed in PD induces a chronic inflammatory response with an increased production of cytokines—IL 1, IL 6, IL 8, which led to increased levels of CRP, a common marker of CVD [18]. On the other hand, in the severe forms of COVID-19 infections, the circulating level of CRP is very high. Moreover, it was observed that systemic inflammation caused by a viral infection is correlated with the platelet activation, thus with an increased risk of MI development [69,70].

On the other hand, it was observed that in patients with PD, the risk of COVID-19 infection is higher. Moreover, the rate of hospital admission and mortality was higher in participants with PD and a concomitant COVID-19 infection [71]. In a larger cohort, in which almost 60.000 participants were followed, it was observed that the risk of COVID-19 infection was not influenced by PD. Interestingly, in obese patients with PD, the hospital admission rate and the mortality was higher compared to those without PD [72].

It was observed that people who express more RACE2 receptors, are more susceptible to develop severe forms of COVID-19. Recent studies revealed the presence of RACE2 at the oral gingiva, tongue and mucosa [73]. In healthy patients, even if the level of RACE2 is extremely expressed, the secreted levels of cytokines are lower. In contrast, in COVID-19 positive patients, even in the case of a lower number of RACE2, the secreted level of cytokines is highly expressed. The same modifications were observed in patients with PD, due to bacterial infections [74]. Therefore, co-infection between COVID-19 and PD leads to more severe forms of disease and may represent a major factor triggering the cytokine storm.

It has been reported that patients with CVD develop an increased risk of severe forms of COVID-19 infection due to circulating levels of miRNA146a [75]. In a study performed on 138 hospitalized patients with COVID-19 by Roganovic et al. (2021), the most frequent risk factors observed were hypertension (31.2%), followed by other CVD (14.5%) [75]. Additionally, it was demonstrated that of the 28 human miRNAs involved in COVID-19 genome, miRNA 146 was involved in immunity modulation. Thus, the virus may affect the miRNA activity and modify the host’s immune response (excessive cytokine production), also accelerating PD and CVD [75]. Furthermore, young patients with COVID-19 infections are more frequently obese (*p* = 0.0002). In a study performed by Zhang et al. (2020), it was observed that CVD (*p* < 0.001) is most frequently associated with COVID-19 infection, followed by respiratory diseases (*p* = 0.003) and inflammatory diseases (*p* = 0.020) [76,77] Patients with comorbidities are more predisposed to develop severe forms of COVID-19 (*p* < 0.001) [77]. Notably, the treatment with glucocorticoids used in COVID-19 infection seems to modulate the miRNA 146 activity [78].

Patients with known coronary artery disease present a higher risk of developing an ACS during acute infections and other acute inflammatory responses [79,80]. In these conditions it is anticipated that patients with known CVDs would be susceptible to a higher risk of ACS and death during the severe inflammatory responses such as the one during COVID-19 infection. Since the underlying CVDs are more often found in the elderly, the adverse cardiovascular events are more prone to appear in this population category than in younger individuals.

Periodontopathic bacteria have been observed to be present in a patient’s metagenome of COVID-19, especially *Prevotella*, *Staphylococcus* and *Fusobacterium* [81]. Additionally, in a series of case reports, it was described that in patients with suspected or confirmed COVID-19 infection, oral modifications were present, including necrotic PD [82,83]. Additionally, it was observed that periodontopathic bacteria are involved in aspiration pneumonia, thus patients with PD present an increased risk of COVID-19 infection worsening and a higher mortality [84]. A similar study, performed by Larvin et al. (2020), revealed that patients with PD present a double risk of mortality if they contract COVID-19, compared to the control group (OR 1.71, 95% CI 1.05–2.72) [71]. In a study published by Marouf et al. (2021), in patients with PD the risk of COVID-19 complications was significantly higher, including intensive care unit admission and death, also an increased blood level of inflammatory markers was observed [85].

## 6. Conclusions

PD, CVD and COVID-19 are linked diseases that share a common pathophysiological substrate represented by inflammation. However, the causality relationship between these entities has not been elucidated thus far. Thus, further studies are still needed to elucidate whether COVID-19 severity and mortality are favored by the inflammatory reactions triggered by PD and atherosclerosis, or all the three diseases are rather manifestations of the systemic inflammation, which links them in a very complex pathophysiological circle.

## Figures and Tables

**Table 1:** Main results of the clinical studies investigating pro-inflammatory cytokines in COVID-19 infection.

Study	Pro-Inflammatory Cytokine	Main Findings	Statistical Relevance
Galvan-Roman J.M. et al. [51]	IL 6	IL6 baseline level are directly correlated with the COVID-19 severity and the necessity of invasive ventilation	*p* < 0.0001
Gao Y. et al. [52]	IL6	IL 6 is an independent risk factor	*p* = 0.005
Liu F. et al. [53]	IL 6	The IL6 level predicts the COVID-19 severity	*p* < 0.001
Coomes E., Haghbayan H. [54]	IL 6	The IL 6 levels are strongly correlated with COVID-19 adverse clinical outcomes	
Xie Y. et al. [55]	IL6	IL 6 level was higher in patients with CVD and COVID-19. Moreover, the higher the IL 6 level, the more severe the COVID -19 form is	*p* < 0.05
Varchetta S. et al. [56]	IL 6	Increased levels of IL 6 were found in serum of COVID-19 patients with severe forms. Moreover, IL 6 level was higher in non-survivor vs. survivors	*p* < 0.0001 *p* < 0.05
Al-Samkari H. et al. [57]	CRP	Elevated CRP level is a predictor of thrombosis in patients with COVID-19	OR 2.71, CI 95%, 1.26–5.86
Huang Y. et al. [58]	CRP	The CRP level was higher in COVID-19 non-survivor patients	*p* < 0.0001
Qin C. et al. [59]	CRP	CRP level is increased in severe vs. non-severe COVID 19 forms	*p* < 0.001
Zhang J.J. et al. [60]	CRP	In severe COVID-19 group CRP levels are higher	*p* < 0.001
Valizadeh H. et al. [61]	Tumor necrosis factor α	In COVID-19 group, NF was higher compared with control group	*p* < 0.0001
Venet F. et al. [62]	Tumor necrosis factor α	Higher values of NF were found in non-survivor vs. survivors	
Hashemian S.R. et al. [63]	Tumor necrosis factor α	Lower levels of NF were associated with survivor status	*p* < 0.01

**Table 2:** In hospital COVID-19 mortality in patients with and without cardiac injury.

Author, Journal, Year	Study Population	Mortality	Association with Myocardial Injury	Mortality in Patients with Myocardial Injury
Huang et al., Lancet, 2020 [64]	*n* = 41	6 (15.00%)	5 (12.20%)	N.A.
Wang et al., JAMA, 2020 [65]	*n* = 138	6 (4.35%)	10 (7.25%)	N.A.
Zhou et al., Lancet, 2020 [66]	*n* = 191	54 (28.27%)	33 (17.28%)	32 (16.75%)
Shi et al., JAMA, 2020 [67]	*n* = 416	57 (13.67%)	82 (19.71%)	42 (10.10%)
Al-Wahaibi et al., SN Compr Clin Med, 2020 [68]	*n* = 143	24 (16.78%)	31 (21.68%)	16 (11.19%)
